# Diagnostic redirection in dementia-first spinocerebellar ataxia type 17: a family-based case report and focused literature review

**DOI:** 10.3389/fnins.2026.1882898

**Published:** 2026-07-13

**Authors:** Siqi Zhang, Xie Zhang, Lijuan Li, Bingling Zhou, Wei Shao

**Affiliations:** 1 First Clinical Medical College, Hubei University of Chinese Medicine, Wuhan, Hubei, China; 2 Department of Neurology, Wuhan No. 1 Hospital/Wuhan Hospital of Traditional Chinese and Western Medicine, Wuhan, Hubei, China

**Keywords:** case report, cerebellar ataxia, dementia, spinocerebellar ataxia type 17, TATA-box binding protein

## Abstract

**Background:**

Spinocerebellar ataxia type 17 (SCA17) is an autosomal dominant repeat-expansion disorder with marked phenotypic heterogeneity. Cognitive and neuropsychiatric symptoms may dominate early recognition and initially suggest a primary dementia syndrome. We aimed to illustrate diagnostic redirection in dementia-first SCA17 through integrated clinical, imaging, familial, and molecular assessment.

**Case presentation:**

We describe the clinical course, neurological findings, cognitive and functional assessments, ancillary investigations, neuroimaging, pedigree information, and molecular genetic findings of a proband with SCA17 and one tested at-risk adult relative. To contextualize the family, we conducted a focused literature review of genetically confirmed SCA17 case and family reports identified through PubMed, Web of Science, Embase, China National Knowledge Infrastructure (CNKI), and Wanfang up to April 16, 2026.

**Findings:**

Repeat-expansion testing established SCA17 in a proband who had initially presented through a dementia-first clinical pathway, with TATA-box binding protein (TBP) alleles of 37/51 repeats. Targeted presymptomatic cascade testing identified the same expanded 51-repeat allele in her asymptomatic adult daughter. Review of 25 published studies showed broad variation in age at onset, TBP repeat size, family context, presenting syndrome, and cumulative phenotype, including cognition-dominant, behavior-dominant, Huntington disease-like, parkinsonian, dystonic, choreic, seizure-associated, and atypical neuroimaging presentations.

**Conclusion:**

The present family illustrates a dementia-first route to SCA17 recognition, in which the initial syndrome-based dementia interpretation remained etiologically provisional as cerebellar signs, cerebellar-predominant atrophy, autosomal-dominant family context, and TBP expansion were integrated. This case-based perspective is intended to support etiological reconsideration in selected dementia-first presentations, rather than to serve as validated clinical criteria.

## 1 Introduction

**Figure 1:**
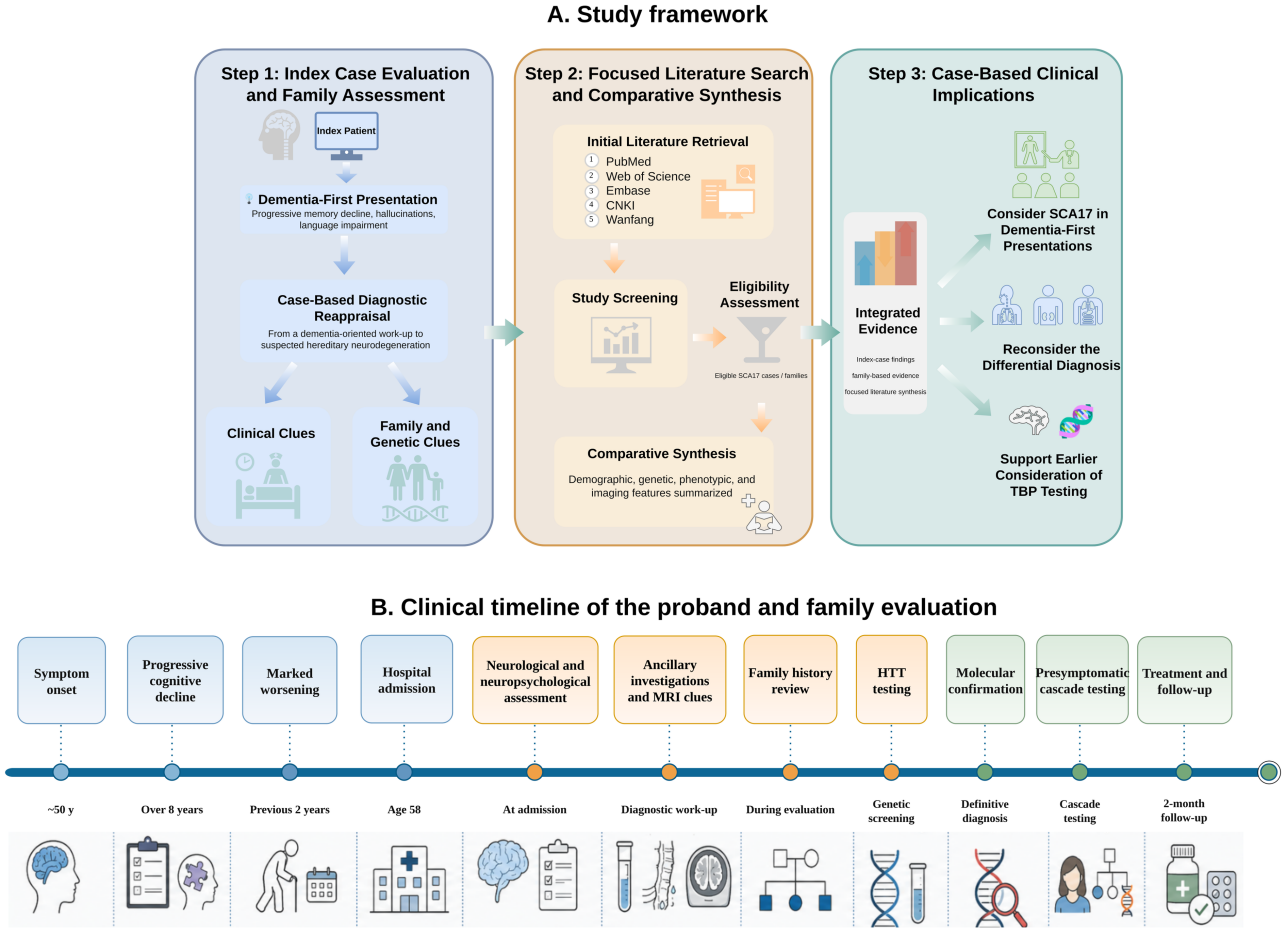
Study framework and clinical timeline of the proband and family evaluation. **(A)** Study framework integrating index-case and family assessment, focused literature review, and case-based clinical implications. **(B)** Clinical timeline from symptom onset at approximately 50 years of age through 8 years of progressive cognitive decline, marked worsening during the preceding 2 years, hospital admission at age 58, neurological and neuropsychological assessment, ancillary investigations and brain magnetic resonance imaging, family-history review, negative huntingtin gene (HTT) repeat-expansion testing, molecular confirmation of spinocerebellar ataxia type 17 by TATA-box binding protein (TBP) repeat-expansion analysis, targeted presymptomatic cascade testing in the asymptomatic adult daughter, treatment, and 2-month follow-up.

Hereditary ataxias are clinically heterogeneous, and some subtypes extend well beyond a predominantly cerebellar syndrome (Coarelli et al., 2023). Among them, spinocerebellar ataxia type 17 (SCA17) is notable for prominent cognitive and psychiatric manifestations (Nethisinghe et al., 2018). In some patients, these features may dominate early clinical recognition and steer evaluation initially toward dementia rather than ataxia (Olszewska et al., 2019). What matters diagnostically, however, is not cognitive-behavioral progression alone, but the emergence of discordant findings that are difficult to reconcile with a conventional dementia work-up and that prompt reconsideration of hereditary neurodegeneration (Olszewska et al., 2019; Nielsen et al., 2012). The practical diagnostic question, therefore, is not whether SCA17 can include cognitive and psychiatric symptoms, but when a dementia-first presentation should be reconsidered as hereditary neurodegeneration despite the absence of an overtly ataxic first impression. We present a family-based case report in which evolving cerebellar findings redirected an initially dementia-oriented diagnostic work-up and led to the diagnosis of SCA17. To contextualize this diagnostic course, we also conducted a focused literature review of genetically confirmed SCA17 cases and families, examining the syndrome-based routes through which SCA17 enters clinical recognition and the cumulative features that may prompt targeted repeat-expansion testing. The overall study framework and the integrated clinical timeline of the proband and family evaluation are presented in Figures 1A,B.

## 2 Methods

### 2.1 Clinical case and family assessment

The clinical course of the proband and the available family information were retrospectively reviewed at the Department of Neurology of our hospital. The collected information included demographic characteristics, disease course, neurological examination findings, laboratory investigations, cerebrospinal fluid studies, dementia-related biomarkers, autoimmune and paraneoplastic antibody testing, neuroimaging findings, treatment, and follow-up information.

Brain magnetic resonance imaging (MRI) was reviewed to evaluate cerebral and cerebellar involvement. Genetic testing was performed on peripheral blood at Guangzhou Oumeng Weiyi Medical Laboratory. The proband underwent HTT repeat analysis and an 11-locus hereditary ataxia repeat-expansion panel covering SCA1, SCA2, SCA3, SCA6, SCA7, SCA8, SCA10, SCA12, SCA17, SCA36, and dentatorubral-pallidoluysian atrophy (DRPLA), using PCR-capillary electrophoresis; apparently homozygous results at selected loci were further assessed by triplet-primed PCR (TP-PCR). A broad hereditary ataxia-related short-read next-generation sequencing assay with bioinformatic analysis of sequence variants and exon-level copy-number changes was also performed. After confirmation of the familial TBP expansion, targeted presymptomatic cascade testing was undertaken in the proband’s adult daughter by PCR-capillary electrophoresis following pre-test genetic counseling, clinical assessment of her psychological readiness and decision-making capacity, and written informed consent. The result was subsequently explained during post-test counseling, and continued neurological follow-up and access to psychological support were arranged. The final diagnosis was based on the expanded TBP allele together with the compatible clinical phenotype, family history, and neuroimaging findings.

### 2.2 Focused literature review

To contextualize the present family-based SCA17 case, we performed a focused literature review rather than a formal systematic review or meta-analysis. This review was used solely to place the present family in clinical context, rather than to function as an independent evidence-generating study, systematic review, meta-analysis, or source of diagnostic criteria. PubMed, Web of Science, Embase, China National Knowledge Infrastructure (CNKI), and Wanfang were searched from database inception to April 16, 2026, using the English terms “spinocerebellar ataxia type 17” and “SCA17,” together with the corresponding Chinese-language terms.

Reports were eligible if they described genetically confirmed SCA17 cases or families with extractable clinical, genetic, and/or neuroimaging information. Reviews without individual case data, duplicate reports, conference abstracts with insufficient clinical detail, non-SCA17 hereditary ataxias, and reports lacking extractable individual- or family-level information were excluded. Extracted variables included demographic features, report type, number of affected individuals, age at onset, TBP repeat size, family history, initial manifestation, ataxia, cognitive impairment, psychiatric or behavioral symptoms, other neurological findings, and neuroimaging features. Because the available literature consisted mainly of case reports, family reports, and small case series, the extracted data were used for qualitative comparison rather than pooled prevalence estimation.

## 3 Results

### 3.1 Clinical presentation and neurological assessment

The proband was a 58-year-old woman who presented with progressive memory decline over 8 years, with marked worsening during the previous 2 years. At symptom onset, she had episodic memory impairment, predominantly affecting recent events, but remained independent in activities of daily living. Early complaints were mainly cognitive, behavioral, and language-related, without prominent gait disturbance or other obvious cerebellar symptoms. As the disease progressed, she became unable to manage money or complete familiar daily tasks, developed disorientation to time and place, and eventually could not find her way home. Language dysfunction gradually became prominent, with impaired expression and comprehension, and her family reported occasional hallucinations. By the year before admission, she could no longer distinguish the front from the back of clothing, could not use a mobile phone or remote control, and had become dependent on full-time family care. Before admission, the family also reported difficulty speaking and understanding others and a tendency to hold food in the mouth without swallowing. There was no clear history of seizures or definite limb motor dysfunction. No obvious loss of smell or taste, limb tremor, bradykinesia, abnormal sleep behaviors, abnormal sweating, or urinary or fecal incontinence was reported. Before admission, she had received no systematic treatment and no etiological dementia diagnosis had been established. The family did not initially report falls or progressive ataxia, but directed questioning elicited slower walking and occasional need for assistance over the preceding 1–2 years.

The initial routine neurological examination documented consciousness, mild dysarthria, unremarkable cranial nerve findings, grade I Kubota water swallowing test, and preserved limb strength. After review of the dementia-oriented investigations and brain MRI, directed reassessment of cerebellar and postural signs showed right finger-to-nose dysmetria, left finger-to-nose clumsiness, wide-based unsteady gait, inability to perform tandem walking, and a positive pull test. Bilateral heel-to-shin testing was poorly completed. A SARA score of 8 was retrospectively estimated from the cerebellar and gait findings documented during this later directed reassessment, rather than obtained from a complete item-level assessment at the initial routine examination. Muscle tone was intermittently mildly increased in the upper limbs, without definite bradykinesia or resting tremor, and the UPDRS-III score was 3. Deep tendon reflexes were symmetric, Babinski signs were absent, and sensory examination was unremarkable.

### 3.2 Ancillary investigations and neuroimaging

Standardized neuropsychological and functional assessment revealed severe global cognitive impairment on the Mini-Mental State Examination (MMSE, 3) and Montreal Cognitive Assessment (MoCA, 1), along with neuropsychiatric symptoms on the Neuropsychiatric Inventory (NPI, 14) and significant impairment in activities of daily living (ADL, 41). Owing to limited resources, more extensive neuropsychological testing was not performed.

All routine laboratory tests, including blood counts, serum biochemistry, and immune tests, were unremarkable. Cerebrospinal fluid studies, including biochemical testing, adenosine deaminase, lactate dehydrogenase, acid-fast staining, and India ink staining, were normal. Cerebrospinal fluid dementia biomarkers, including amyloid-*β* 1–42 (Aβ1–42), amyloid-β 1–40 (Aβ1–40), the Aβ1–42/Aβ1–40 ratio, phosphorylated tau at threonine 181 (p-tau181), and total tau, were also within normal ranges. Additional testing, including anti-N-methyl-D-aspartate receptor (NMDAR) antibodies, a paraneoplastic neurological syndrome antibody panel, ceruloplasmin, dynamic positional testing, head-shaking testing, spontaneous nystagmus assessment, and orthostatic blood pressure measurement, was unremarkable.

**Figure 2:**
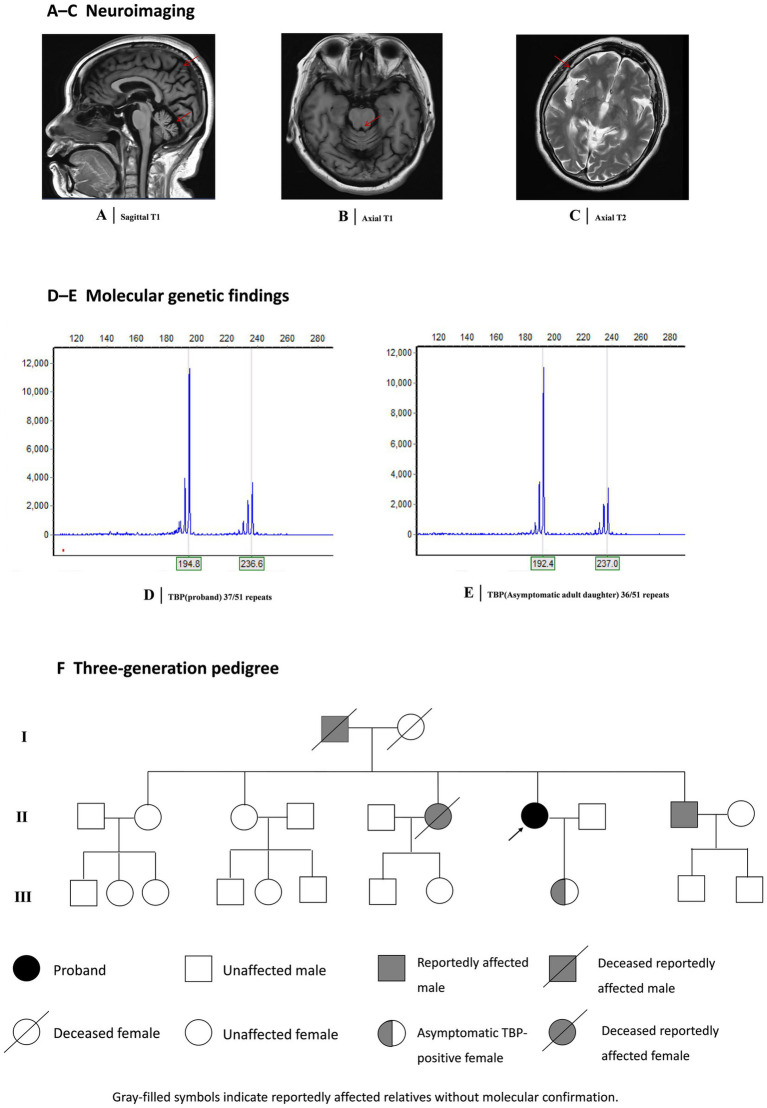
Neuroimaging, molecular genetic findings, and pedigree of the SCA17 family. **(A–C)** Brain magnetic resonance imaging of the proband. Sagittal T1-weighted **(A)**, axial T1-weighted **(B)**, and axial T2-weighted **(C)** images demonstrate global cerebral atrophy with relatively prominent cerebellar atrophy and deep white matter ischemic changes corresponding to Fazekas grade II. Red arrows indicate representative regions of cerebral and cerebellar atrophy. **(D)** TATA-box binding protein (TBP) repeat-length analysis in the proband shows alleles of 37 and 51 repeats, confirming spinocerebellar ataxia type 17. **(E)** Targeted presymptomatic cascade testing in the asymptomatic adult daughter shows TBP alleles of 36 and 51 repeats, demonstrating transmission of the expanded 51-repeat allele. **(F)** Three-generation pedigree of the family. The arrow indicates the proband; squares and circles represent males and females, respectively; open symbols indicate relatives without reported neurological involvement; gray symbols indicate reportedly affected relatives; the half-filled circle indicates the asymptomatic adult daughter carrying the expanded TBP allele; and diagonal lines indicate deceased individuals. Gray symbols denote relatives with family-reported progressive cognitive/language decline and later loss of independence; the first symptom and cognitive–ataxic sequence could not be reliably reconstructed.

Brain MRI demonstrated global cerebral atrophy, most pronounced in the cerebellum, with deep white matter ischemic changes (Fazekas grade II; Figures 2A–C). Imaging studies revealed no significant abnormalities on B-mode ultrasound of the liver, gallbladder, spleen, pancreas, and adrenal glands. Color Doppler ultrasound showed normal post-void residual volume, and no Kayser–Fleischer rings were observed.

### 3.3 Genetic confirmation and family verification

Family-history information was obtained retrospectively, and no medical records or neurological examinations were available for the reportedly affected relatives. The proband’s father, one elder sister, and younger brother were reported to have progressive cognitive decline with language impairment, followed by later loss of independence; the reported ages at onset were approximately 70, 40, and 48 years, respectively. The father and elder sister were unavailable for genetic testing because they had died before the molecular diagnosis was established; the sister died at 55 years of age. The younger brother did not undergo TBP testing because geographical and financial barriers prevented evaluation at our center. Because the family history was retrospective, the exact first symptom and the temporal relationship between cognitive decline and gait or ataxic manifestations could not be reliably reconstructed. The proband’s 34-year-old daughter had no clinical symptoms at the time of evaluation and subsequently underwent genetic testing.

Molecular evaluation of the proband included HTT repeat analysis, an 11-locus hereditary ataxia repeat-expansion panel, and a broad hereditary ataxia-related next-generation sequencing assay with exon-level copy-number variation analysis. HTT alleles contained 17 and 18 CAG repeats and were within the normal range. The repeat-expansion panel identified TBP alleles of 37 and 51 repeats (Figure 2D), whereas no pathogenic expansion was identified at the other tested loci. The next-generation sequencing assay did not identify an additional pathogenic or likely pathogenic variant that could explain the phenotype, and no clinically relevant exon-level copy-number variant was detected. Together with the compatible clinical phenotype, neuroimaging findings, and autosomal-dominant family context, the pathogenic-range 51-repeat TBP allele established the diagnosis of SCA17. Targeted presymptomatic cascade testing in the adult daughter identified TBP alleles of 36 and 51 repeats (Figure 2E). Pedigree analysis supported an autosomal-dominant inheritance pattern across three generations (Figure 2F).

After diagnosis, symptomatic treatment with donepezil, memantine, tanshinone, oxiracetam, and vitamin B12 was initiated. At the 2-month follow-up, the family reported that the patient could perform simple household tasks, such as hanging clothes, and had milder behavioral symptoms, with no obvious further cognitive deterioration perceived. No repeat standardized cognitive or neuropsychiatric assessment was performed.

### 3.4 Focused literature review findings

The literature search yielded 316 records, and 25 genetically confirmed studies were included after duplicate removal, screening, and full-text assessment. These reports consisted mainly of genetically confirmed single-case reports, family reports, and small case series.

Across the included literature, the 25 studies supported multiple syndrome-based entry routes into SCA17 recognition rather than a single ataxia-centered pathway. Clinical attention could begin through cognitive or behavioral decline, progressive ataxia, Huntington disease-like chorea or dystonia, parkinsonian or multiple system atrophy-like presentations, seizure-associated presentations, or atypical neuroimaging patterns. These entry routes were variable, but the cumulative phenotype often converged on combinations of cerebellar, cognitive-behavioral, extrapyramidal, pyramidal, bulbar, ocular motor, seizure-related, and imaging abnormalities. These reports were used to contextualize the present family within the known phenotypic variability of SCA17, rather than to define SCA17-specific diagnostic criteria or differentiation rules.

## 4 Discussion

The present family illustrates how the evolving multisystem phenotype of SCA17 can redirect an initially dementia-oriented diagnostic work-up. The proband entered clinical evaluation through a dementia-first pathway, but the cumulative evidence—objective cerebellar signs, cerebellar-predominant atrophy, autosomal-dominant family context, negative HTT testing, and a pathogenic TBP expansion—shifted the diagnostic focus toward hereditary repeat-expansion neurodegeneration. The focused synthesis of 25 genetically confirmed reports provides clinical context for this interpretation: SCA17 may enter recognition through multiple syndrome-based routes, whereas diagnostic clarification depends on integrating clinical, imaging, familial, and molecular evidence.

### 4.1 Genetic and familial context

The clinicogenetic characteristics and family contexts of the 25 included studies are summarized in Table 1. Published SCA17 cases were reported predominantly as single cases, family reports, or small case series. Age at onset varied widely across studies, although most patients presented in adulthood, particularly from early middle age to late adulthood. TBP repeat sizes were likewise variable, ranging approximately from 41 to 66 repeats in the included reports. Most cases showed familial aggregation (Olszewska et al., 2019; De Michele et al., 2003), although sporadic cases or cases with unclear family history were also described (Bech et al., 2010; Hire et al., 2011; Guo et al., 2025).

**Table 1:** Clinicogenetic characteristics, diagnostic-entry features, and major neuroimaging findings of published SCA17 cases and families.

Study	Country/ethnicity	Report type	No. affected	Age at onset	TBP repeat size	Family history	Initial manifestation/diagnostic entry	Main cumulative phenotype	Main neuroimaging findings
Loy et al. (2005)	United Kingdom	single case	1	42 y	47 repeats	No	Dysarthria; cerebellar/bulbar entry	Gait ataxia with cognitive impairment, chorea, and dystonia	Cerebellar atrophy; putaminal rim hyperintensity
Hagenah et al. (2004)	German family	family report	3	20–45 y	53–55 repeats	Yes	Dystonia; movement-disorder entry	Ataxia with dysarthria, spasticity/dysphagia, and cognitive involvement in later disease	Variable: normal MRI to mild cerebellar/cerebral atrophy
Zhang et al. (2013)	China	family report	4	39–55 y	45 repeats	Yes	Memory decline; cognitive/dementia-first entry	Mild gait/limb ataxia with memory decline and dysarthria	Cerebellar and cerebral atrophy
De Michele et al. (2003)	Italy	family series	11	3–53 y	NR	Yes	Psychiatric/cognitive symptoms; psychiatric/behavioral entry	Cerebellar ataxia with dementia, behavioral change, dystonia, dysphagia, and seizures	Cerebral and cerebellar atrophy
Maltecca et al. (2003)	Italy	family report	4	3–35 y	53–66 repeats	Yes	Ataxia/gait instability; ataxia/gait entry	Gait/limb ataxia with early cognitive impairment, behavioral symptoms, dystonia, dysphagia, and seizures	Cerebral and cerebellar atrophy; pontine T2 hyperintensity
Bech et al. (2010)	Denmark	single case	1	25 y	54 repeats	No	Memory decline; cognitive/dementia-first entry	Gait/limb ataxia with cognitive impairment, dystonia, parkinsonism, and pyramidal signs	Global, brainstem, and cerebellar atrophy; putaminal rim hyperintensity
Hire et al. (2011)	India	case series	3	19–41 y	47–48 repeats	Yes	Gait instability; ataxia/gait entry	Gait/limb ataxia with cognitive impairment, behavioral change, dysarthria/dysphagia, and parkinsonism	Variable cerebellar degeneration or cerebral/cerebellar atrophy
Tremolizzo et al. (2011)	Italy	single case	1	23 y	43 repeats	No	Ataxia/gait instability; ataxia/gait entry	Gait/limb ataxia with cognitive impairment, dysarthria, dysphagia, and seizures	Cerebral/pontocerebellar atrophy in the proband; isolated cerebellar atrophy in some carriers
Nielsen et al. (2012)	Denmark	single case	1	42 y	43 repeats	Yes	Cognitive decline; cognitive/dementia-first entry	Gait ataxia with cognitive impairment, behavioral change, dysarthria, tremor, and pyramidal signs	Mild cerebellar hemispheric/vermian atrophy; cerebellar hypometabolism
Koutsis et al. (2014)	Greece	family report	3	22–28 y	54 repeats	Yes	Gait instability; ataxia/gait entry	Gait/limb ataxia with cognitive impairment, behavioral change, chorea, dystonia, and seizures	Cerebellar atrophy with mild brainstem/cerebral atrophy
Olszewska et al. (2019)	Ireland	family report	5	32–50 y	43 repeats	Yes	Behavioral changes; behavioral/FTD-like entry	Frontal executive dysfunction with cerebellar/ataxic features, apathy, dysarthria, and spasticity	Cerebellar/vermian atrophy; cerebellar hypometabolism
Mariotti et al. (2007)	Italy	case series	15	19–55 y	44–53 repeats	Yes	Variable; ataxia/HD-like referral entry	Gait/limb ataxia with cognitive impairment, chorea, dystonia, parkinsonism, and pyramidal signs	Cerebral and cerebellar atrophy; mild brainstem atrophy in some patients
Origone et al. (2018)	Italy	single case	1	54 y	41 repeats	Yes	Depression/personality change; psychiatric/behavioral entry	Gait/limb ataxia with cognitive impairment, behavioral change, dysarthria/dysphagia, and chorea	Frontoparietal and cerebellar atrophy; cerebellar/putaminal hypometabolism
Berns et al. (2026)	Chinese descent	single case	1	4 y	43 repeats	NR	Seizures/developmental delay; early-onset complex entry	Cerebellar ataxia with cognitive involvement, dystonia, choreoathetosis, and seizures	Initially normal MRI; later mesial temporal sclerosis without cerebellar atrophy
Paparella et al. (2025)	Italy	single case	1	53 y	41 repeats	No	Chorea; HD-like movement-disorder entry	Chorea with depressive symptoms and only minimal gait uncertainty	CT: no SCA-specific abnormality; mild chronic white-matter changes/slight cortical atrophy; MRI unavailable
Grassini et al. (2024)	Italy	single case	1	39 y	52 repeats	Yes	Behavioral changes; cognitive-behavioral entry	Gait/limb ataxia with cognitive impairment, apathy/irritability, dysarthria, dysphagia, and pyramidal signs	Frontal, hippocampal, cerebellar, and brainstem atrophy; atypical FDG-PET pattern
Mehanna and Itin (2013)	German	single case	1	70 y	44 repeats	Yes	Gait instability; rapidly progressive ataxia entry	Rapidly progressive gait ataxia with frontal executive dysfunction and tremor	Normal MRI
Salvatore et al. (2006)	Italy	family series	4	23–40 y	51–53 repeats	Yes	Gait instability/depression; ataxia-parkinsonian entry	Gait/limb ataxia with cognitive impairment, depression, dystonia, dysphagia, and parkinsonism	Variable: slight cerebellar atrophy to cerebral/cerebellar atrophy; abnormal DAT-SPECT in advanced cases
Wagle Shukla et al. (2023)	White	single case	1	NR	43 repeats	No	Dystonia/tremor; movement-disorder entry	Generalized dystonia and arm tremor, followed later by dysarthria and gait/limb ataxia	Normal MRI at initial workup
Zühlke et al. (2005)	German family	family report	2	41–50 y	52–53 repeats	Yes	Gait instability; ataxia/gait entry	Gait/limb ataxia with dysarthria	Cortical cerebellar atrophy; mildly reduced brain volume
Liu et al. (2016)	China	family report	2	12–33 y	52–55 repeats	Yes	Gait instability/memory decline; ataxia-cognitive entry	Gait/limb ataxia with cognitive impairment, behavioral change, parkinsonism, chorea, and dystonia	Variable brain, cerebellar, hippocampal, and brainstem atrophy
Liang et al. (2019)	China	single case	1	42 y	42 repeats	No	Parkinsonism; parkinsonian/MSA-like entry	Gait/limb ataxia with dysarthria, pyramidal signs, and ocular motor abnormalities	Generalized, cerebellar, and brainstem atrophy; possible hot cross bun sign
Wu et al. (2026)	China	single case	1	51 y	42 repeats	No	Gait instability; ataxia/gait entry	Gait/limb ataxia with dysarthria, pyramidal signs, and ocular motor abnormalities	Cerebellar atrophy; white-matter ischemic lesions; reduced right cerebellar perfusion
Reetz et al. (2011)	Germany	case series	16	18–47 y	44–55 repeats	NR	NR; cohort imaging study	Cerebellar ataxia with cognitive impairment in a SCA17 cohort	Predominant bilateral cerebellar atrophy on VBM
Guo et al. (2025)	China	single case	1	53 y	43 repeats	No	Gait instability; MSA-like/ataxia entry	Gait/limb ataxia with dysphagia, parkinsonism, and pyramidal signs	Cerebellar and pontine atrophy; hot cross bun sign

CT, computed tomography; DAT-SPECT, dopamine transporter single-photon emission computed tomography; FDG-PET, fluorodeoxyglucose positron emission tomography; FTD, frontotemporal dementia; HD, Huntington disease; MRI, magnetic resonance imaging; MSA, multiple system atrophy; No., number; NR, not reported; SCA17, spinocerebellar ataxia type 17; T2, T2-weighted imaging; TBP, TATA-box binding protein; VBM, voxel-based morphometry; y, years. Country or ethnicity was extracted as reported in the original articles and was used only to describe the geographic and familial context of published SCA17 reports.

Taken together, the published literature indicates that TBP repeat size and family context define genetic susceptibility, but not a fixed clinical entry pattern (Bech et al., 2010; Guo et al., 2025; Zühlke et al., 2005). The predominance of familial cases supports careful pedigree assessment and cascade testing (Olszewska et al., 2019; Tremolizzo et al., 2011), whereas sporadic cases or cases with unclear family history indicate that the absence of an obvious pedigree does not exclude SCA17 (Bech et al., 2010; Guo et al., 2025; Paparella et al., 2025). Family history should therefore not be treated as a passive background variable, but as an active diagnostic anchor when cognitive, behavioral, movement-disorder, or ataxic features remain difficult to reconcile with a primary sporadic dementia framework.

### 4.2 Diagnostic-entry routes and cumulative phenotype

Table 2 organizes the published cases by syndrome-based diagnostic-entry routes rather than by isolated symptoms. This structure highlights a clinically important dissociation between the presenting syndrome and the cumulative phenotype of SCA17. The first feature that brings a patient to clinical attention may be cognitive decline, behavioral change, chorea, dystonia, parkinsonism, seizure, or ataxia, but the diagnostic signal often emerges only after these features are interpreted together with cerebellar signs, imaging abnormalities, family context, and repeat-expansion testing.

**Table 2:** Reported syndrome-based routes into SCA17 recognition in the literature.

Syndrome-based entry route	Representative reports	Later convergent features	Contextual relevance
Cognitive/dementia-first	Zhang et al. (2013); Nielsen et al. (2012); present family	Cognitive decline accompanied by cerebellar signs, cerebellar atrophy, family history, and TBP expansion	Provides context for keeping the dementia label provisional when cerebellar or familial features emerge
Behavioral/FTD-like	Olszewska et al. (2019); De Michele et al. (2003); Grassini et al. (2024)	Behavioral change, executive dysfunction, psychiatric symptoms, ataxia, and TBP expansion	Provides context for etiological reconsideration in familial or atypical FTD-like presentations
HD-like/chorea-dystonia	Loy et al. (2005); Mariotti et al. (2007); Hagenah et al. (2004); Paparella et al. (2025)	Chorea, dystonia, ataxia, cognitive impairment, and negative HTT testing where reported	Provides context for considering targeted TBP testing in selected HTT-negative HD-like presentations
Parkinsonian/MSA-like	Liang et al. (2019); Guo et al. (2025); Salvatore et al. (2006)	Parkinsonism, autonomic or pontocerebellar features, cerebellar atrophy, and TBP expansion	Provides context for syndrome-level overlap with sporadic degenerative movement disorders
Seizure/early-onset complex	Maltecca et al. (2003); Tremolizzo et al. (2011); Berns et al. (2026)	Seizures with ataxia, cognitive decline, dystonia, or multisystem neurological involvement	Provides context for considering repeat-expansion testing in selected complex early-onset multisystem presentations

FTD, frontotemporal dementia; HD, Huntington disease; HTT, huntingtin gene; MSA, multiple system atrophy; SCA17, spinocerebellar ataxia type 17; TBP, TATA-box binding protein.

Published SCA17 cases suggest that the initial manifestations can be grouped into several syndrome-based entry routes rather than a single ataxia-centered pathway. Zhang et al. (2013) described a family in which the proband first noticed memory decline, with only mild ataxia despite marked cerebellar and cerebral atrophy; Nielsen et al. (2012) reported rapidly progressive cognitive decline with only subtle cerebellar signs before SCA17 was established. Behavioral or psychiatric entries are even better documented: Olszewska et al. (2019) reported an autosomal-dominant frontotemporal dementia (FTD)-like family in which a microtubule-associated protein tau (MAPT) variant failed to segregate with disease, whereas a 43-repeat TBP expansion did; De Michele et al. (2003) similarly noted that behavioral symptoms and frontal impairment may precede ataxia, rigidity, and dystonic movements. Movement-disorder entries form another major route. Mariotti et al. (2007) identified SCA17-positive patients among both progressive ataxia and Huntington-like referrals, Hagenah et al. (2004) reported focal dystonia as the presenting sign in all affected members of a German family, and Paparella et al. (2025) described an almost isolated chorea-dominant presentation with a 41-repeat TBP allele. Seizures were less often a stand-alone entry route, although the childhood-onset case reported by Berns et al. (2026) shows that epilepsy may complicate early recognition in complex early-onset SCA17. The diagnostic risk is therefore not simply missing ataxia, but accepting the first syndromic label before family history, cerebellar signs, neuroimaging, and repeat-expansion testing are integrated.

Against this misleading entry pattern, the later clinical profile shows a more informative convergence. Ataxia was a recurrent feature across reports, most often documented as gait ataxia, limb ataxia, or cerebellar ataxia, making cerebellar involvement a more stable cumulative feature than a uniform presenting symptom (Hire et al., 2011; Mariotti et al., 2007). Cognitive and behavioral involvement was also recurrently reported, but its significance depends on the broader neurological context rather than its isolated presence (De Michele et al., 2003; Mariotti et al., 2007; Koutsis et al., 2014). Similarly, bulbar, extrapyramidal, pyramidal, ocular motor, tremor-related, and seizure-related manifestations do not define SCA17 individually, but they support a multisystem neurodegenerative interpretation when they occur alongside ataxia and cognitive–behavioral involvement (De Michele et al., 2003; Hire et al., 2011; Mariotti et al., 2007; Koutsis et al., 2014). Thus, the diagnostic value lies in the convergence of cerebellar, cognitive–behavioral, and multisystem neurological features rather than in any single symptom.

The present case exemplifies this pattern: a dementia-oriented presentation was redirected toward SCA17 only when objective cerebellar signs, relatively prominent cerebellar atrophy, autosomal-dominant family history, and the expanded TBP allele were integrated. Rather than defining a novel phenotype, this case illustrates how the expected evolution of a multisystem neurodegenerative disorder can prompt diagnostic redirection once the cumulative clinicogenetic and imaging profile becomes apparent.

### 4.3 Neuroimaging clues and diagnostic limitations

Neuroimaging findings in SCA17 vary across reports, but the variation is not entirely diffuse. The most reproducible abnormality is cerebellar atrophy, frequently accompanied by cerebral or cortical atrophy. This pattern is supported by both larger imaging-based studies and case reports: Mariotti et al. (2007) reported cortical and cerebellar atrophy in all 15 patients in their series, and Reetz et al. (2011) demonstrated predominant bilateral cerebellar volume loss on voxel-based morphometry, together with an association between cerebellar atrophy and CAG repeat length. Other family-based and single-case reports further described extension to the cerebellar vermis, pontocerebellar structures, pons, or brainstem (Guo et al., 2025; Tremolizzo et al., 2011; Koutsis et al., 2014; Origone et al., 2018; Liu et al., 2016). However, the degree of structural abnormality does not reliably parallel the clinical foreground or apparent disease severity. Marked cerebellar or cerebral atrophy may be present despite relatively mild clinical findings, whereas severe cognitive, ataxic, or hyperkinetic presentations have also been reported with mild, normal, or nonspecific structural imaging (Nielsen et al., 2012; Zhang et al., 2013; Berns et al., 2026; Mehanna and Itin, 2013; Wagle Shukla et al., 2023). MRI therefore provides anatomical context, but it cannot be used as a linear surrogate for clinical stage or severity.

Extracerebellar abnormalities help explain why SCA17 may be misread as other neurodegenerative syndromes. Basal ganglia signal changes, striatal or sensorimotor metabolic abnormalities, and nigrostriatal dopaminergic dysfunction may support Huntington disease-like or parkinsonian impressions (Bech et al., 2010; Loy et al., 2005; Grassini et al., 2024; Salvatore et al., 2006). Pontine or pontocerebellar involvement, including occasional hot cross bun-like changes, may suggest a multiple system atrophy (MSA)-like process, whereas frontal, hippocampal, cortical, or cerebellar metabolic abnormalities may reinforce a dementia- or frontotemporal dementia-like diagnostic pathway (Olszewska et al., 2019; Guo et al., 2025; Grassini et al., 2024; Liang et al., 2019). None of these findings is specific for SCA17. Their importance is that, when combined with cerebellar involvement and the clinical phenotype, they broaden rather than narrow the diagnostic field. Accordingly, cerebellar-predominant atrophy remains the most useful radiological clue, extracerebellar findings account for syndrome-level overlap, and normal or nonspecific imaging does not exclude SCA17.

In the present case, brain MRI showed global cerebral atrophy with relatively prominent cerebellar atrophy, consistent with the cerebellar-predominant pattern identified in the reviewed reports and making a purely dementia-oriented interpretation insufficient. The accompanying deep white matter ischemic changes are better regarded as nonspecific age- or vascular-related findings than as central SCA17-related abnormalities.

### 4.4 Differential diagnosis in dementia-first and HD-like presentations

At presentation, the clinical picture reasonably overlapped with a dementia syndrome, given the progressive cognitive decline, hallucinations, language disturbance, and impaired comprehension. In this context, dementia was regarded as the presenting syndrome rather than as an exclusive etiological diagnosis. The diagnostic question was therefore not whether the patient had dementia, but whether a conventional dementia-oriented framework could account for the complete neurological, imaging, familial, and molecular profile. That interpretation became less satisfactory once the patient was reassessed as a broader neurological syndrome: dysarthria, dysmetria, broad-based gait, a compatible family history, and cerebellar-predominant atrophy were not readily explained by a conventional dementia framework, particularly after dementia-oriented and secondary-cause investigations were unrevealing. With normal HTT repeat testing, the differential diagnosis shifted toward HTT-negative Huntington disease-like disorders and hereditary ataxia–dementia syndromes.

Previous SCA17 reports provide two useful parallels for the diagnostic course in this family. Olszewska et al. (2019) described an autosomal-dominant FTD-like family in which a MAPT variant initially appeared plausible but did not segregate with disease, whereas a 43-repeat TBP expansion did. De Michele et al. (2003) similarly showed that behavioral change and frontal impairment may dominate early SCA17 and precede ataxia, rigidity, and dystonic movements, making diagnosis of a spinocerebellar ataxia difficult. In the present family, the early cognitive-behavioral and language syndrome therefore fit a dementia-oriented work-up, but this explanation became less satisfactory once cerebellar signs, relatively prominent cerebellar atrophy, autosomal-dominant inheritance, and TBP expansion were considered together. A second parallel comes from the HTT-negative Huntington disease-like spectrum. Loy et al. (2005) reported a Huntington disease-like SCA17 case with normal HTT repeats, in which dentatorubral-pallidoluysian atrophy (DRPLA) and other chorea–ataxia disorders were considered before TBP expansion established the diagnosis. Mariotti et al. (2007) also identified SCA17-positive patients among both progressive ataxia and Huntington-like referrals, supporting TBP testing when Huntington disease testing is negative. In the present patient, normal HTT repeats argued against Huntington disease, and the absence of myoclonus, epilepsy, or definite choreoathetosis made a DRPLA-like disorder less fitting. The pathogenic 51-repeat TBP allele, together with the cerebellar, familial, and imaging findings, provided the more coherent explanation.

### 4.5 Molecular diagnostic approach and limitations

Although the pathogenic TBP expansion was confirmed by PCR-capillary electrophoresis, the genetic work-up did not rely on single-gene testing alone. The proband also underwent an 11-locus repeat-expansion panel and broader short-read next-generation sequencing with bioinformatic analysis of sequence variants and exon-level copy-number changes. These methods provide complementary information: locus-specific PCR-based assays remain important reference and confirmatory methods for established repeat expansions, whereas short-read sequencing can broaden the search for additional genetic causes and, when coupled with dedicated repeat-expansion callers, can simultaneously screen multiple known repeat-expansion loci (Ibañez et al., 2022). However, short-read approaches may not fully resolve the length and internal sequence structure of large or complex repeat expansions (Stevanovski et al., 2022). Targeted long-read sequencing may further extend the diagnostic scope by directly characterizing repeat length, motif composition, haplotype context, and internal sequence interruptions (Stevanovski et al., 2022). These approaches were not available in the present case. Therefore, although the molecular evaluation was broader than targeted TBP testing, it was not exhaustive. Because no additional reportedly affected relative underwent molecular testing beyond the proband and her asymptomatic adult daughter, formal co-segregation of the TBP expansion with the clinical phenotype could not be assessed across the wider family. Nevertheless, the full-penetrance-range 51-repeat TBP allele, together with the proband’s compatible phenotype and neuroimaging findings, provides strong molecular support for SCA17 in the proband (Rossi et al., 2023).

### 4.6 Clinical implications

The clinical implication is not that TBP testing should be broadly applied to all patients with cognitive decline. Rather, this family illustrates a narrower clinical context in which diagnostic reconsideration is warranted: a dementia-first presentation accompanied by unrevealing conventional dementia-oriented and secondary-cause investigations, objective cerebellar signs, cerebellar-predominant atrophy, and an autosomal-dominant family history. In this setting, the diagnostic focus may appropriately shift from primary dementia toward hereditary repeat-expansion neurodegeneration, making TBP repeat analysis a targeted rather than general dementia-screening test. Thus, the value of the present case lies in syndromic integration: cognitive decline, subtle cerebellar signs, cerebellar-predominant atrophy, and an autosomal-dominant family context were considered together rather than interpreted as isolated or mutually exclusive diagnostic labels.

Targeted cascade testing extended the clinical-translational relevance of the diagnosis. Identification of the same expanded TBP allele in the proband’s asymptomatic adult daughter established her carrier status and placed the family within a heritable risk framework. This supports genetic counseling, risk assessment, and structured longitudinal follow-up for at-risk relatives.

This family-based case report and focused literature review suggest that the diagnostic challenge of SCA17 lies less in phenotypic breadth itself than in the separation between clinical entry and diagnostic clarification. A dementia-like presentation should prompt diagnostic reconsideration when accumulating evidence points beyond a primary dementia framework and toward hereditary repeat-expansion neurodegeneration. In the present case, diagnostic redirection refers to etiological reassessment during the index hospitalization, rather than correction of a previously established dementia diagnosis or an externally documented diagnostic delay. The practical value of this perspective lies in illustrating when targeted TBP repeat testing and family-level risk assessment may become appropriate. This interpretation should be regarded as a case-based diagnostic perspective rather than a novel diagnostic construct or a validated diagnostic algorithm, and future multicenter registries or prospective longitudinal cohorts are needed to refine its applicability and clarify clinicogenetic relationships.

## Data Availability

De-identified data supporting the findings of this report are available from the corresponding author upon reasonable request, subject to applicable ethical and privacy restrictions.
