# Supplementary material for: Diagnostic redirection in dementia-first spinocerebellar ataxia type 17: a family-based case report and focused literature review
Source: Front Neurosci. 2026 Jul 13;20:1882898. doi: 10.3389/fnins.2026.1882898 (PMC13402550; doi:10.3389/fnins.2026.1882898)
Supplement: Supplementary file 1 [file Table_1.docx]

**Supplementary Table 1.** Detailed presenting symptoms, cumulative phenotypes, and neuroimaging findings of published SCA17 cases and families

| **Study** | **Initial manifestation** | **Ataxia** | **Cognitive impairment/dementia** | **Psychiatric/behavioral symptoms** | **Other neurological findings** | **Neuroimaging findings** |
| --- | --- | --- | --- | --- | --- | --- |
| Loy, 2005^9^ | dysarthria | gait ataxia | cognitive impairment | NR | dysarthria, chorea, dystonia | cerebellar atrophy; putaminal rim hyperintensity |
| Hagenah, 2004^10^ | dystonia | gait and limb ataxia | cognitive impairment | behavioral changes | dysarthria, spasticity, dysphagia | variable: normal MRI to mild cerebellar/cerebral atrophy |
| Zhang, 2013^11^ | memory decline | gait and limb ataxia | memory decline | NR | dysarthria | cerebellar and cerebral atrophy |
| De Michele, 2003^5^ | psychiatric symptoms | cerebellar ataxia | dementia | behavioral changes | dystonia, dysphagia, seizures | cerebral and cerebellar atrophy |
| Maltecca, 2003^12^ | gait instability | gait and limb ataxia | dementia | behavioral changes | dystonia, dysphagia, seizures | cerebral and cerebellar atrophy; pontine T2 hyperintensity |
| Bech, 2010^6^ | memory decline | gait and limb ataxia | cognitive impairment | NR | dystonia, parkinsonism, pyramidal signs | global, brainstem, and cerebellar atrophy; putaminal rim hyperintensity |
| Hire, 2011^7^ | gait instability | gait and limb ataxia | cognitive impairment | behavioral changes | dysarthria, dysphagia, parkinsonism | variable: cerebellar degeneration or cerebral/cerebellar atrophy |
| Tremolizzo, 2011^13^ | gait instability | gait and limb ataxia | cognitive impairment | NR | dysarthria, dysphagia, seizures | variable: cerebral and ponto-cerebellar atrophy; isolated cerebellar atrophy in some carriers |
| Nielsen, 2012^4^ | cognitive decline | gait ataxia | cognitive impairment | behavioral changes | dysarthria, tremor, pyramidal signs | mild cerebellar hemispheric/vermian atrophy; cerebellar hypometabolism |
| Koutsis, 2014^14^ | gait instability | gait and limb ataxia | cognitive impairment | behavioral changes | chorea, dystonia, seizures | variable: cerebellar atrophy with mild brainstem/cerebral atrophy |
| Olszewska, 2019^3^ | behavioral changes | gait ataxia | frontal executive dysfunction | apathy | dysarthria, spasticity | variable: cerebellar/vermian atrophy; cerebellar hypometabolism |
| Mariotti, 2007^15^ | gait instability | gait and limb ataxia | cognitive impairment | behavioral changes | chorea, dystonia, parkinsonism | cerebral and cerebellar atrophy; mild brainstem atrophy |
| Origone, 2018^16^ | depression | gait and limb ataxia | cognitive impairment | depression, behavioral changes | dysarthria, dysphagia, chorea | frontoparietal and cerebellar atrophy; brainstem sparing; cerebellar/putaminal hypometabolism |
| Berns, 2026^17^ | seizures | cerebellar ataxia | cognitive impairment | NR | dystonia, chorea, seizures | normal MRI initially; later mesial temporal sclerosis without cerebellar atrophy |
| Paparella, 2025^18^ | chorea | mild gait ataxia | NR | depression | chorea | slight cortical atrophy and mild white matter ischemic changes on CT; MRI unavailable |
| Grassini, 2024^19^ | behavioral changes | gait and limb ataxia | cognitive impairment | apathy, irritability | dysarthria, dysphagia, pyramidal signs | frontal, hippocampal, cerebellar, and brainstem atrophy; atypical FDG-PET pattern |
| Mehanna, 2013^20^ | gait instability | gait ataxia | frontal executive dysfunction | NR | tremor | normal MRI |
| Salvatore, 2006^21^ | gait instability | gait and limb ataxia | cognitive impairment | depression | dystonia, dysphagia, parkinsonism | variable: slight cerebellar atrophy to cerebral/cerebellar atrophy; abnormal DAT-SPECT in advanced cases |
| Wagle Shukla, 2023^22^ | dystonia | gait and limb ataxia | NR | depression | tremor, dysarthria, dystonia | normal MRI at initial workup |
| Zühlke, 2005^23^ | gait instability | gait and limb ataxia | NR | NR | dysarthria | cortical cerebellar atrophy; mildly reduced brain volume |
| Liu, 2016^24^ | gait instability | gait and limb ataxia | cognitive impairment | behavioral changes | parkinsonism, chorea, dystonia | variable within family: brain, cerebellar, hippocampal, and brainstem atrophy |
| Liang, 2019^25^ | parkinsonism | gait and limb ataxia | NR | NR | dysarthria, pyramidal signs, ocular movement abnormalities | generalized, cerebellar, and brainstem atrophy; possible hot cross bun sign |
| Wu, 2026^26^ | gait instability | gait and limb ataxia | NR | NR | dysarthria, pyramidal signs, ocular movement abnormalities | cerebellar atrophy; white matter ischemic lesions; reduced right cerebellar perfusion |
| Reetz, 2011^27^ | NR | cerebellar ataxia | cognitive impairment | NR | NR | predominant bilateral cerebellar atrophy on VBM |
| Guo, 2025^8^ | gait instability | gait and limb ataxia | NR | NR | dysphagia, parkinsonism, pyramidal signs | cerebellar and pontine atrophy; hot cross bun sign |

**Note:** CT, computed tomography; DAT-SPECT, dopamine transporter single-photon emission computed tomography; FDG-PET, fluorodeoxyglucose positron emission tomography; MRI, magnetic resonance imaging; NR, not reported; SCA17, spinocerebellar ataxia type 17; T2, T2-weighted imaging; VBM, voxel-based morphometry.
